# Pneumococcal colonization in older adults

**DOI:** 10.1186/s12979-016-0057-0

**Published:** 2016-01-12

**Authors:** Susanna Esposito, Daniela Mari, Luigi Bergamaschini, Annalisa Orenti, Leonardo Terranova, Luca Ruggiero, Valentina Ierardi, Monia Gambino, Francesco Della Croce, Nicola Principi

**Affiliations:** Department of Pathophysiology and Transplantation, Pediatric Highly Intensive Care Unit, Università degli Studi di Milano, Fondazione IRCCS Ca’ Granda Ospedale Maggiore Policlinico, Via Commenda 9, 20122 Milan, Italy; Department of Medical Sciences and Community Health, Geriatric Unit, Università degli Studi di Milano, Milan, Italy; Department of Biomedical and Clinical Sciences “Luigi Sacco”, Università degli Studi di Milano, Milan, Italy; Department of Clinical Sciences and Community Health, Laboratory of Medical Statistics, Epidemiology and Biometry “G.A. Maccacaro”, Università degli Studi di Milano, Milan, Italy; ASP, Pio Albergo Trivulzio, Milan, Italy

**Keywords:** Invasive pneumococcal disease, Pneumococcal carriage, Pneumococcal colonization, 13-valent pneumococcal conjugate vaccine, 23-valent polysaccharide pneumococcal vaccine, 13-valent pneumococcal vaccine, Pneumococcal vaccination, Prevention, *Streptococcus pneumoniae*

## Abstract

**Background:**

Little is known about pneumococcal carrier states in older adults. The main aim of this study was to evaluate pneumococcal colonization patterns among older adults in two centres in Milan, Italy, before the widespread use of the 13-valent pneumococcal vaccine (PCV13) in this age group, to investigate demographic and clinical features that are associated with pneumococcal colonization and to estimate the potential coverage offered by PCV13.

**Results:**

Among 417 adults ≥65 years old (171, 41.1 %, ≥75 years), 41 (9.8 %) were pneumococcal carriers. Univariate and multivariate analyses revealed that pneumococcal colonization was significantly less common among individuals with underlying co-morbidities than among those without (odds ratio [OR] 0.453, 95 % confidence interval [CI] 0.235–0.875, *p* = 0.018; adjusted OR 0.503, 95 % CI 0.255–0.992, *p* = 0.047). Moreover, among these patients, those with cardiac disease had a significantly lower risk of colonization (OR 0.308, 95 % CI 0.119–0.795, *p* = 0.015; adjusted OR 0.341, 95 % CI 0.13–0.894, *p* = 0.029). Only one vaccinated subject who received 23-valent polysaccharide pneumococcal vaccine (PPV23) was colonized. Twenty-five (89.3 %) of the subjects who were <75 years old and 9 (75.0 %) of those who were ≥75 years old were colonized by at least one of the serotypes that is included in PCV13, with serotype 19 F being the most common. Respiratory allergies as well as overall co-morbidities were more common in subjects who were positive for only non-PCV13 serotypes compared with negative subjects and those who were carriers of only PCV13 serotypes.

**Conclusions:**

Although this study included a relatively small number of subjects and has been performed in a limited geographic setting, results showed that pneumococcal colonization in older people is common, and the monitoring of carriers can offer useful information about the circulation of this pathogen among older people and the potential protective effect of pneumococcal vaccines. Because the colonization in most cases involves the strains that are included in PCV13, this vaccine could be useful in the prevention of pneumococcal infections in the overall population of older people. In subjects with respiratory allergies and in those with co-morbidities, the addition of the PPV23 to PCV13 should be recommended. Due to the low vaccination coverage, urgent educational programmes are required to inform older adults and their medical doctors of the risks of pneumococcal infection and the efficacy and safety of the available pneumococcal vaccines.

## Background

The clinical relevance of *Streptococcus pneumoniae* in older adults has been highlighted for several years [1]. Effective vaccines and antibiotics to prevent and treat pneumococcal infection have been developed and used worldwide in clinical practice. This development has led to a significant reduction in pneumococcal diseases and has provided great benefit from medical, social and economic viewpoints [2]. Nevertheless, *S. pneumoniae* remains a leading cause of severe infectious diseases, with its highest incidence in children < 2 years old and in people ≥65 years old [1, 2]. The incidence of invasive pneumococcal disease (IPD) is estimated to be 10-20/100,000/year in developed countries [3, 4] and significantly higher in the third world [5]. Moreover, pneumonia causes millions of deaths annually in older people [6, 7]. This circumstance implies that further attempts to reduce the total burden of pneumococcal infection must be made.

Because pharyngeal pneumococcal carriage is considered to be a prerequisite for the development of pneumococcal infections and a basis for the diffusion of the pathogen [8], the monitoring of pneumococcal carriage can offer relevant information about the risk of the development of the disease, not only for the carrier but also for the community. Moreover, because the administration of pneumococcal conjugate vaccines can significantly influence colonization in both vaccinated and unvaccinated subjects [9], the evaluation of the characteristics of carriage can be extremely useful in evaluating the vaccine’s impact and its potential direct and indirect effects. Most of the studies in this regard were conducted with young children. In these subjects, it has been established that colonization rates could be as high as 75 % and pneumococcal conjugate vaccine administration leads to a reduction in the incidence of infections due to the pneumococcal serotypes included in the vaccine, in both vaccinated and unvaccinated subjects, mainly through the impact on carriage [10]. However, little is known about the carrier state among older adults. Knowledge in this regard appears to be important because of the growing immunological pressure due to the increasing vaccine coverage in children, the availability of the 23-valent polysaccharide pneumococcal vaccine (PPV23), and the recent introduction of the 13-valent pneumococcal vaccine (PCV13) for adults [11]. The main aim of this study was to evaluate the pneumococcal colonization pattern among older adults in two centres in Milan, Italy, before the widespread use of PCV13 in this age group, to investigate the demographic and clinical features that are associated with pneumococcal colonization and to estimate the potential coverage offered by PCV13.

## Results

Table 1 summarizes the general characteristics of the 417 (302, 72.4 %, females; 246, 58.9 %, <75 years old) enrolled subjects according to their pneumococcal colonization status. Of these subjects, 41 (9.8 %) were pneumococcal carriers using the most effective methods for evaluating pneumococcal colonization (i.e., oropharyngeal instead of nasopharyngeal sampling, molecular methods instead of non-enriched cultures, amplification of both the *lytA* and *cpsA* genes instead of l*ytA* gene only, nylon fibre tips instead of Dacron and ryon swabs) [12–18]. Univariate and multivariate analyses revealed that pneumococcal colonization was significantly less common among individuals who had underlying co-morbidities compared with those without (odds ratio [OR] 0.453, 95 % confidence interval [CI] 0.235–0.875, *p* = 0.018; adjusted OR 0.503, 95 % CI 0.255–0.992, *p* = 0.047). Moreover, among these patients, those with cardiac disease had a significantly lower risk of colonization (OR 0.308, 95 % CI 0.119–0.795, *p* = 0.015; adjusted OR 0.341, 95 % CI 0.13–0.894, *p* = 0.029). By contrast, none of the remaining studied variables were significantly associated with pneumococcal colonization; these studied variables were gender, ethnicity, number and type of co-habitants, marital status, smoking habit, presence of respiratory allergy, influenza vaccination in the previous season, mean number of respiratory infections in the previous six months, hospitalization and admission to the Emergency Room in the previous 3 months, and mean number of pneumococcal infections in the previous month. Interestingly, although there were no statistically significant differences, individuals <75 years old was more common among pneumococcal carriers than among negative subjects (68.3 % vs 58.0 %; *p* = 0.205; adjusted *p* = 0.306), whereas antibiotic use in the previous month was more frequent among negative subjects than among pneumococcal carriers (12.8 % vs 2.4 %; *p* = 0.084; adjusted *p* = 0.069). Moreover, only one subject who was vaccinated with pneumococcal vaccine and received PPV23 was colonized, and due to the limited number of vaccinated subjects, it was not possible to evaluate the impact of pneumococcal vaccines on carriers. Although the association is not statistically significant, it is worthwhile to note that previous pneumococcal vaccination reduced the risk of pneumococcal colonization (OR 0.323, 95 % CI 0.043–2.44, *p* = 0.273; adjusted OR 0.389, 95 % CI 0.051–2.969, *p* = 0.362).

**Table 1:** Characteristics of the study population and association analysis with pneumococcal colonization

		Total *n* = 417	Negative *n* = 376	Positive *n* = 41	Odds ratio (95 % CI)	*p*-value	Adjusted odds ratio (95 % CI)	*p*-value
Age	<75 years	246 (58.99)	218 (57.98)	28 (68.29)	(reference)		(reference)	
	≥75 years	171 (41.01)	158 (42.02)	13 (31.71)	0.641 (0.322–1.276)	0.205	0.685 (0.332–1.414)	0.306
Mean age (sd)		73.97 (6.66)	74.13 (6.64)	72.49 (6.76)	0.961 (0.911–1.013)	0.135	0.969 (0.917–1.025)	0.277
Sex	Male	115 (27.58)	106 (28.19)	9 (21.95)	(reference)		(reference)	
	Female	302 (72.42)	270 (71.81)	32 (78.05)	1.396 (0.644–3.023)	0.398	1.208 (0.549–2.658)	0.638
Ethnicity	Caucasian	403 (96.64)	366 (97.34)	37 (90.24)	(reference)		(reference)	
	Non-Caucasian	10 (2.4)	8 (2.13)	2 (4.88)	2.473 (0.506–12.078)	0.263	1.862 (0.37–9.384)	0.451
	NA	4 (0.96)	2 (0.53)	2 (4.88)				
Mean number of cohabitants (sd)		0.22 (0.55)	0.23 (0.56)	0.11 (0.39)	0.563 (0.228–1.393)	0.214	0.441 (0.152–1.275)	0.131
Children cohabitants	No	401 (96.16)	361 (96.01)	40 (97.56)				
	Yes	2 (0.48)	2 (0.53)	0 (0)	NE	NE	NE	NE
	NA	14 (3.36)	13 (3.46)	1 (2.44)				
Nephew	No	195 (46.76)	179 (47.61)	16 (39.02)	(reference)		(reference)	
	Yes	89 (21.34)	82 (21.81)	7 (17.07)	0.955 (0.378–2.41)	0.922	0.856 (0.325–2.253)	0.753
	NA	133 (31.89)	115 (30.59)	18 (43.9)				
Marital status	Married/Cohabiting	223 (53.48)	200 (53.19)	23 (56.1)	(reference)		(reference)	
	Single	57 (13.67)	52 (13.83)	5 (12.2)	0.836 (0.303–2.305)	0.729	0.652 (0.214–1.982)	0.451
	Separated	53 (12.71)	47 (12.5)	6 (14.63)	1.11 (0.428–2.879)	0.83	1.129 (0.429–2.971)	0.806
	Widowed	83 (19.9)	76 (20.21)	7 (17.07)	0.801 (0.33–1.943)	0.623	0.994 (0.395–2.503)	0.99
	NA	1 (0.24)	1 (0.27)	0 (0)				
Smoker	No	367 (88.01)	331 (88.03)	36 (87.8)	(reference)		(reference)	
	Yes	49 (11.75)	44 (11.7)	5 (12.2)	1.045 (0.389–2.803)	0.931	0.78 (0.261–2.331)	0.657
	NA	1 (0.24)	1 (0.27)	0 (0)				
Respiratory allergy	No	291 (69.78)	265 (70.48)	26 (63.41)	(reference)		(reference)	
	Yes	126 (30.22)	111 (29.52)	15 (36.59)	1.377 (0.703–2.7)	0.351	1.208 (0.602–2.424)	0.596
Co-morbidities	No	137 (32.85)	117 (31.12)	20 (48.78)	(reference)		(reference)	
	Yes	278 (66.67)	258 (68.62)	20 (48.78)	0.453 (0.235–0.875)	**0.018**	0.503 (0.255–0.992)	**0.047**
	NA	2 (0.48)	1 (0.27)	1 (2.44)				
Group of co-morbidities	Respiratory	86 (20.62)	79 (21.01)	7 (17.07)	0.518 (0.209–1.284)	0.156	0.613 (0.236–1.59)	0.314
	Cardiac	120 (28.78)	114 (30.32)	6 (14.63)	0.308 (0.119–0.795)	**0.015**	0.341 (0.13–0.894)	**0.029**
	Metabolic	23 (5.52)	20 (5.32)	3 (7.32)	0.877 (0.238–3.229)	0.844	0.945 (0.255–3.51)	0.933
	Tumours	10 (2.4)	10 (2.66)	0 (0)	NE	NE	NE	NE
	Thyroid	23 (5.52)	20 (5.32)	3 (7.32)	0.877 (0.238–3.229)	0.844	0.848 (0.23–3.13)	0.805
	Other	16 (3.84)	15 (3.99)	1 (2.44)	0.39 (0.049–3.119)	0.375	0.448 (0.055–3.639)	0.453
	None	137 (32.85)	117 (31.12)	20 (48.78)	(reference)		(reference)	
	NA	2 (0.48)	1 (0.27)	1 (2.44)				
Pneumococcal vaccination	No	389 (93.29)	349 (92.82)	40 (97.56)	(reference)		(reference)	
	Yes	28 (6.71)	27 (7.18)	1 (2.44)	0.323 (0.043–2.44)	0.273	0.389 (0.051–2.969)	0.362
Type of vaccination	PCV13	10 (2.4)	10 (2.66)	0 (0)				
	PPV23	17 (4.08)	16 (4.26)	1 (2.44)	NE	NE	NE	NE
	NA	390 (93.53)	350 (93.09)	40 (97.56)				
Influenza vaccination in the previous season	No	215 (51.56)	191 (50.8)	24 (58.54)	(reference)		(reference)	
	Yes	202 (48.44)	185 (49.2)	17 (41.46)	0.731 (0.38–1.406)	0.348	0.84 (0.419–1.682)	0.623
Mean number of respiratory infections in the previous 6 months (sd)		0.9 (1.87)	0.89 (1.92)	0.97 (1.32)	1.022 (0.863–1.211)	0.798	0.998 (0.827–1.203)	0.981
Mean number of upper respiratory tract in the previous 6 months (sd)		0.61 (1.64)	0.6 (1.67)	0.74 (1.29)	1.046 (0.874–1.251)	0.625	1.005 (0.817–1.235)	0.966
Mean number of lower respiratory tract infections in the previous 6 months (sd)		0.28 (0.68)	0.29 (0.69)	0.22 (0.48)	0.823 (0.45–1.504)	0.526	0.899 (0.49–1.646)	0.729
Hospitalizations in the last 3 months	No	404 (96.88)	363 (96.54)	41 (100)				
	Yes	13 (3.12)	13 (3.46)	0 (0)	NE	NE	NE	NE
Emergency Room visit in the last 3 months	No	388 (93.05)	352 (93.62)	36 (87.8)	(reference)		(reference)	
	Yes	24 (5.76)	21 (5.59)	3 (7.32)	1.397 (0.397–4.912)	0.602	1.651 (0.46–5.928)	0.442
	NA	5 (1.2)	3 (0.8)	2 (4.88)				
Treatments with antibiotic in the last month	No	368 (88.25)	328 (87.23)	40 (97.56)	(reference)		(reference)	
	Yes	49 (11.75)	48 (12.77)	1 (2.44)	0.171 (0.023–1.271)	**0.084**	0.155 (0.021–1.158)	**0.069**
Pneumococcal infections in the last month	No	363 (87.05)	326 (86.7)	37 (90.24)	(reference)		(reference)	
	Yes	54 (12.95)	50 (13.3)	4 (9.76)	0.705 (0.241–2.063)	0.523	0.606 (0.204–1.802)	0.368

Odds ratios, with pertinent 95 % CIs and *p*-values, are obtained by logistic regression. Adjustment is made for age and the presence of co-morbidity, which are potentially confounding factors

*95 % CI* 95 % confidence interval, *NA* not available, *NE* not executable *OR* odds ratio, *PCV13* 13-valent pneumococcal vaccine, *PPV23* 23-valent polysaccharide pneumococcal vaccine, *SD* standard deviation

Bold data indicate significant differences

In one out of 41 subjects with pneumococcal colonization, serotyping was not possible. Table 2 summarizes the serotypes that were carried by the 40 individuals for whom serotyping was possible according to age group. Twenty-five (89.3 %) of the subjects who were <75 years old and 9 (75.0 %) of those who were ≥75 years old were colonized by at least one of the serotypes included in PCV13. In both groups, the serotype 19 F was the most common carried serotype (85.7 and 66.7 % in subjects who were <75 years old and ≥75 years old, respectively). However, in six individuals, the serotypes that were included only in the PCV13 were identified. Serotypes that were not included in PCV13 were evidenced in 23 (82.1 %) of the subjects <75 years old and 11 (91.7 %) of those ≥75 years old. The most common non-PCV13 serotypes were in both groups, serotypes 24 and 15 (53.6 % and 35.7 % vs 75.0 % and 41.7 % in subjects who were <75 years old and ≥75 years old, respectively). Globally, serotypes that were not included in PCV13 were included in PPV23 in 55.7 % of the cases. Six patients were positive only for non-PCV13 serotypes. None of them was colonized only by serotypes that were included in PPV-23.Table 3 reports the characteristics of the subjects who were enrolled in the study, divided by the positivity for the serotypes. Interestingly, respiratory allergies (66.7 % vs 29.4 %, 0.0 %, and 40.0 %, *p* = 0.073, adjusted *p* = 0.086) as well as overall co-morbidities (100.0 % vs 68.7 %) and cardiac underlying diseases (50.0 % vs 21.2 %) were more common in subjects who were positive only for non-PCV13 serotypes compared with negative subjects, those carriers of only PCV13 serotypes. By contrast, no significant difference was observed from comparing negative and positive subjects regardless of the serotypes as well as comparing the three groups of positive subjects with regard to age distribution, gender, number and type of co-habitants, marital status, smoking habit, previous pneumococcal vaccination, influenza vaccination in the previous season, mean number of respiratory infections in the last six months, hospitalization and admission to the Emergency Room in the previous 3 months, antibiotic treatment in the last month, and mean number of pneumococcal infections in the previous month.

**Table 2:** Colonization by specific pneumococcal serotypes according to age group

Positive for serotype	Total (*n* = 40)	Age < 75 (*n* = 28)	Age ≥ 75 (*n* = 12)	OR (95 % IC)
1	4 (10.0)	0 (0.0)	4 (33.33)	NE
3	1 (2.5)	1 (3.57)	0 (0.0)	NE
4	7 (17.5)	5 (17.86)	2 (16.67)	0.92 (0.152–5.566)
6A	2 (5.0)	2 (7.14)	0 (0.0)	NE
9	4 (10.0)	3 (10.71)	1 (8.33)	0.758 (0.071–8.118)
19A	1 (2.5)	1 (3.57)	0 (0.0)	NE
19 F	32 (80.0)	24 (85.71)	8 (66.67)	0.333 (0.067–1.652)
PCV13 serotypes	34 (85)	25 (89.29)	9 (75.0)	0.36 (0.061–2.119)
8	2 (5.0)	2 (7.14)	0 (0.0)	NE
11	2 (5.0)	2 (7.14)	0 (0.0)	NE
12	5 (12.5)	5 (17.86)	0 (0.0)	NE
15	15 (37.5)	10 (35.71)	5 (41.67)	1.286 (0.322–5.13)
21	1 (2.5)	1 (3.57)	0 (0.0)	NE
22	6 (15.0)	5 (17.86)	1 (8.33)	0.418 (0.043–4.024)
24	24 (60.0)	15 (53.57)	9 (75.0)	2.6 (0.578–11.687)
33	4 (10.0)	2 (7.14)	2 (16.67)	2.6 (0.321–21.047)
35B	1 (2.5)	1 (3.57)	0 (0.0)	NE
35 F	1 (2.5)	1 (3.57)	0 (0.0)	NE
Non-PCV13 serotypes	34 (85.0)	23 (82.14)	11 (91.67)	2.391 (0.249–23.009)

*95 % CI* 95 % confidence interval, *NE* not executable, *OR* odds ratio

**Table 3:** Characteristics of patients in the study, divided by positivity for serotypes

		Total *n* = 417	Negative *n* = 376	Positive only for PCV13 serotypes *n* = 6	Positive for PCV13 and non-PCV13 serotypes *n* = 28	Positive only for non-PCV13 serotypes *n* = 6
Age	<75 years	246 (58.99)	218 (57.82)	5 (83.33)	20 (71.43)	3 (50)
	> = 75 years	171 (41.01)	159 (42.18)	1 (16.67)	8 (28.57)	3 (50)
Mean age (sd)		73.97 (6.66)	74.18 (6.7)	69.83 (3.92)	72.32 (6.57)	72.5 (5.01)
Sex	Male	115 (27.58)	106 (28.12)	2 (33.33)	4 (14.29)	3 (50)
	Female	302 (72.42)	271 (71.88)	4 (66.67)	24 (85.71)	3 (50)
Ethnicity	Caucasian	403 (96.64)	367 (97.35)	6 (100)	24 (85.71)	6 (100)
	Non-Caucasian	10 (2.4)	8 (2.12)	0 (0)	2 (7.14)	0 (0)
	NA	4 (0.96)	2 (0.53)	0 (0)	2 (7.14)	0 (0)
Mean number of cohabitants (sd)		401 (96.16)	362 (96.02)	6 (100)	27 (96.43)	6 (100)
Children cohabitants	No	0.22 (0.55)	0.23 (0.56)	0 (0)	0.12 (0.44)	0.17 (0.41)
	Yes	2 (0.48)	2 (0.53)	0 (0)	0 (0)	0 (0)
	NA	14 (3.36)	13 (3.45)	0 (0)	1 (3.57)	0 (0)
Nephew	No	195 (46.76)	180 (47.75)	1 (16.67)	11 (39.29)	3 (50)
	Yes	89 (21.34)	82 (21.75)	1 (16.67)	4 (14.29)	2 (33.33)
	NA	133 (31.89)	115 (30.5)	4 (66.67)	13 (46.43)	1 (16.67)
Marital status	Married/Cohabiting	223 (53.48)	200 (53.05)	4 (66.67)	15 (53.57)	4 (66.67)
	Single	57 (13.67)	52 (13.79)	1 (16.67)	4 (14.29)	0 (0)
	Separated	53 (12.71)	48 (12.73)	1 (16.67)	3 (10.71)	1 (16.67)
	Widowed	83 (19.9)	76 (20.16)	0 (0)	6 (21.43)	1 (16.67)
	NA	1 (0.24)	1 (0.27)	0 (0)	0 (0)	0 (0)
Smoker	No	367 (88.01)	332 (88.06)	5 (83.33)	25 (89.29)	5 (83.33)
	Yes	49 (11.75)	44 (11.67)	1 (16.67)	3 (10.71)	1 (16.67)
	NA	1 (0.24)	1 (0.27)	0 (0)	0 (0)	0 (0)
Respiratory allergies	No	291 (69.78)	266 (70.56)	6 (100)	17 (60.71)	2 (33.33)
	Yes	126 (30.22)	111 (29.44)	0 (0)	11 (39.29)	4 (66.67)
Co-morbidities	No	137 (32.85)	117 (31.03)	3 (50)	17 (60.71)	0 (0)
	Yes	278 (66.67)	259 (68.7)	3 (50)	10 (35.71)	6 (100)
	NA	2 (0.48)	1 (0.27)	0 (0)	1 (3.57)	0 (0)
Group of co-morbidities	Respiratory	86 (20.62)	80 (21.22)	0 (0)	3 (10.71)	3 (50)
	Cardiac	120 (28.78)	114 (30.24)	1 (16.67)	4 (14.29)	1 (16.67)
	Metabolic	23 (5.52)	20 (5.31)	1 (16.67)	1 (3.57)	1 (16.67)
	Tumours	10 (2.4)	10 (2.65)	0 (0)	0 (0)	0 (0)
	Thyroid	23 (5.52)	20 (5.31)	1 (16.67)	2 (7.14)	0 (0)
	Other	16 (3.84)	15 (3.98)	0 (0)	0 (0)	1 (16.67)
	None	137 (32.85)	117 (31.03)	3 (50)	17 (60.71)	0 (0)
	NA	2 (0.48)	1 (0.27)	0 (0)	1 (3.57)	0 (0)
Pneumococcal vaccination	No	389 (93.29)	350 (92.84)	6 (100)	28 (100)	5 (83.33)
	Yes	28 (6.71)	27 (7.16)	0 (0)	0 (0)	1 (16.67)
Type of vaccination	PCV13	10 (2.4)	10 (2.65)	0 (0)	0 (0)	0 (0)
	PPV23	17 (4.08)	16 (4.24)	0 (0)	0 (0)	1 (16.67)
	NA	390 (93.53)	351 (93.1)	6 (100)	28 (100)	5 (83.33)
Influenza vaccination	No	215 (51.56)	191 (50.66)	4 (66.67)	16 (57.14)	4 (66.67)
	Yes	202 (48.44)	186 (49.34)	2 (33.33)	12 (42.86)	2 (33.33)
Mean number of respiratory infections (sd)		0.9 (1.87)	0.89 (1.91)	1.4 (1.14)	0.92 (1.04)	1 (2.45)
Mean number of upper respiratory tract infections (sd)		0.61 (1.64)	0.6 (1.67)	1 (1.22)	0.65 (0.98)	1 (2.45)
Mean number of lower respiratory tract infections (sd)		0.28 (0.68)	0.29 (0.69)	0.4 (0.55)	0.24 (0.52)	0 (0)
Hospitalizations in the last 3 months	No	404 (96.88)	364 (96.55)	6 (100)	28 (100)	6 (100)
	Yes	13 (3.12)	13 (3.45)	0 (0)	0 (0)	0 (0)
Emergency Room visit in the last 3 months	No	388 (93.05)	352 (93.37)	5 (83.33)	25 (89.29)	6 (100)
	Yes	24 (5.76)	22 (5.84)	0 (0)	2 (7.14)	0 (0)
	NA	5 (1.2)	3 (0.8)	1 (16.67)	1 (3.57)	0 (0)
Treatments with antibiotic in the last month	No	368 (88.25)	329 (87.27)	6 (100)	27 (96.43)	6 (100)
	Yes	49 (11.75)	48 (12.73)	0 (0)	1 (3.57)	0 (0)
Pneumococcal infections in the last month	No	363 (87.05)	327 (86.74)	6 (100)	24 (85.71)	6 (100)
	Yes	54 (12.95)	50 (13.26)	0 (0)	4 (14.29)	0 (0)

*NA* not available, *PCV13* 13-valent pneumococcal vaccine, *PPV23* 23-valent polysaccharide pneumococcal vaccine, *SD* standard deviation

## Discussion

In this study in which the pneumococcal carriage of subjects who were ≥65 years old was evaluated in two centres in Milan, Italy, a prevalence of 9.3 % was found. Most of the studies that have evaluated the prevalence of pneumococcal carriage in adults have been conducted in subjects who were younger than those included in this research. Consequently, the comparison is difficult because the carriage prevalence in adults can vary widely, according to the methods that are used to identify the pathogen, behavioural issues, the composition of households, contact with children, antibiotic use and vaccine coverage in the paediatric population. Before pneumococcal conjugate vaccine introduction, in most of the studies conducted in the developed world, colonization rates were found to vary between 1.5 and 30 % [19–23]. However, higher values were evidenced in developing countries such as Nigeria and Thailand, where prevalences of 26 and 30 % were found [24, 25]. After the introduction of pneumococcal conjugate vaccines into the immunization schedule of infants and children, a prevalence of 13.8 % was found in Native American adults ≥65 years old [26], approximately 20 % in adults ≥45 years old in Alaska [27], and more than 30 % in adults 60–89 years old in The Netherlands [28]. However, it is likely that these findings do not truly represent the prevalence of pneumococcal carriage in older adults because they included parents of neonates, infants and young children, which introduces a relevant bias due to exposure to the paediatric reservoir. Moreover, as in the case of the study by Krone et al. [28], swabs were collected from adults with influenza-like illnesses. A lower prevalence of carriage was reported by Ansaldi et al. in Italy a few years ago [29]. These authors evaluated the carriage prevalence in a group of adults ≥60 years old who were randomly selected among the general population and living in a geographical area that has had a high pneumococcal vaccination coverage of children for several years. They obtained four different nasopharyngeal samples at a 1-month interval and found that the point prevalence of carriage adjusted for age was stable between the first and fourth time point, being 9.5, 10.7, 7.6 and 10.9 % [29]. The data collected with our study are similar and appear to indicate the real prevalence of pneumococcal carriage in the older population. Oropharyngeal sampling was used to collect the pharyngeal secretions because it has recently been shown that it is a significantly better means of detecting colonization by *S. pneumoniae* than the nasopharyngeal sampling used in previous studies of younger children [12], adolescents [13], and young adults [14]. In addition, *S. pneumoniae* was identified by means of molecular methods which, albeit with some exceptions [15], have been found to be significantly more reliable than the traditional non-enriched cultures used in routine practice [16]. Furthermore, to improve the detection of *S. pneumoniae* without increasing the risk of false-positive results, both the *lytA* and *cpsA* genes were amplified [14], and only the samples in which both the genes were detected in two out of three consecutive tests of the same swab were considered to be positive. This approach avoided false-positive results because only *S. pneumoniae* has both of these genes, whereas different streptococci that are not capsulated but that could colonize the oropharynx possess only the *lytA* gene and not the *cpsA* gene [17]. Finally, previous studies have shown that flocked nylon fibre tip ensures the highest rate of detection of *S. pneumoniae*, particularly compared with the more widely used Dacron and rayon swabs [18]. However, in contrast to what has been evidenced by Ansaldi et al. [29], in this study, no higher prevalence of pneumococcal colonization was found in subjects who were ≥75 years old. Moreover, the presence of an underlying medical condition, mainly cardiac disease and older age were associated with a lower prevalence of pneumococcal colonization. These findings are difficult to explain, and further studies are needed to clarify the problem, although a tendency toward higher prescriptions of antibiotics in patients with co-morbidities and in older patients could explain the results. Indeed, pneumococcal colonization was lower in subjects who were treated with a recent course of antibiotics.

Regarding pneumococcal serotypes, this study showed that almost all of the subjects who were colonized by *S. pneumoniae* were carrying a strain that was included in PCV13, which suggests a potential positive effect of vaccination with this preparation. PCV13 administration is associated with a significant reduction in the pharyngeal carriage of the serotypes that are included in the vaccine, which leads to a significant reduction in the risk of development of pneumococcal disease due to this serotype [8]. Interestingly, it was evidenced that the most common PCV13 serotype found in this study was 19 F, which is a serotype that is included in the 7-valent pneumococcal conjugate vaccine (PCV7) and that should not have been circulated considering the high PCV7 vaccination coverage that has been reached in children for several years in the area where the survey was performed [6]. However, recent studies have shown that some years after PCV7 vaccination, the protective effect against colonization wanes due to the decline in antibody levels, and previously vaccinated children are re-colonized by the same serotypes that were included in the vaccine [30]. Because high antibody levels are needed to avoid colonization by serotype 19 F, a large number of older children become carriers of this serotype and spread it into the community, which favours its colonization in older adults [31]. The majority of our study population was colonized by serotypes included in PCV13, which highlights that PCV13 could have a relevant effect in the protection of older adults. However, considering that the most common non-PCV13 serotypes that were carried by older people were among the most common cause of IPD as evidenced by recent epidemiological studies conducted in Europe after the introduction of PCV13 [32], continued monitoring is needed to evaluate their importance in the older population. This arrangement could be of particular interest in patients with respiratory allergy and in those with co-morbidities who in this study were found to have the highest prevalence of this type of colonization. However, the real importance of PPV23 in the prevention of infections that were potentially due to serotypes included in this vaccine and not in PCV13 is difficult to evaluate from the data collected with this study because in contrast to PCV13, PPV23 does not modify pneumococcal carriage [33]. Nevertheless, PPV23 can prevent IPD because the vaccine included serotypes that could represent an effective measure in case some of the carried serotypes would reach the blood stream. This scenario explains why recent recommendations on the use of pneumococcal vaccination in adults indicate that older patients should receive the PPV23 in addition to PCV13 [34]. Unfortunately, the low pneumococcal vaccination coverage in our population did not permit us to analyse the impact of pneumococcal vaccines on carriage in older adults. However, this limited use of pneumococcal vaccines highlights the urgent need for an educational programme on pneumococcal prevention, which should involve older adults and their medical doctors.

## Conclusions

Although this study included a relatively small number of subjects and has been performed in a limited geographic setting, results showed that pneumococcal colonization in older people is common and that the monitoring of carriers can offer useful information about circulation of the pathogen among older people and the potential protective effect of pneumococcal vaccines. Due to colonization, in most of the cases with strains included in PCV13, the use of this vaccine could be useful in the prevention of pneumococcal infections in the overall population of older people. In subjects with respiratory allergy and in those with co-morbidities, the addition of PPV23 to PCV13 should also be recommended to reduce the risk of IPD. Due to the low vaccination coverage, urgent educational programmes are required to inform older adults and their medical doctors on the risks of pneumococcal infection and the efficacy and safety of the available pneumococcal vaccines. Moreover, further studies are needed to evaluate the possible impact of serotype replacement after the universal use of PCV13 in children and older adults.

## Methods

### Enrolment of patients and swab collection

The study was conducted in Milan, Italy, from March 1, 2015, to July 31, 2015 and two centres for older adults, the University of the Third Age and the Pio Albergo Trivulzio, were involved. The first is a centre in which retired and semi-retired people come together and learn together, not for qualifications but for their own reward. The second is a centre that is specifically devoted to the diagnosis and therapy of geriatric medical problems. The study was approved by the Ethics Committee of both centres and by that of the Fondazione IRCCS Ca’ Granda Ospedale Maggiore Policlinico, Milan, Italy and the Pio Albergo Trivulzio, Milan, Italy.

Subjects aged ≥65 years old who regularly attended the centres for university lessons or periodical medical controls were screened and enrolled. Those with an active respiratory infection and/or who were receiving antibiotics in the 10 days before sampling were excluded from the study. The subjects were enrolled after written informed consent had been obtained. After enrolment, data regarding the medical history, including the pneumococcal vaccination status, were collected on each subject and were recorded in an electronic file that was specifically prepared for the study. Particular attention was paid to medical events that occurred in the six months before enrolment.

The oropharyngeal samples were obtained by trained physicians using an ESwab kit that contained a polypropylene screw-cap tube filled with 1 mL of liquid Amies medium (Brescia, Copan, Italy). The sampling was conducted by pressing the tongue downward to the floor of the mouth with a spatula and swabbing both tonsillar arches and the posterior nasopharynx, without touching the sides of the mouth. All of the swabs were immediately transported to the central laboratory and processed within 2 h of arrival.

### Identification of *S. pneumoniae*

Bacterial genomic DNA was extracted from the samples using a NucliSENS easyMAG automated extraction system (BioMeriéux, Bagno a Ripoli, Florence, Italy), a 250-μL sample input and a generic protocol. The DNA was analysed for the autolysin-A-encoding gene (l*ytA*) and the wzg (*cpsA*) gene of *S. pneumoniae* by real-time polymerase chain reaction (PCR), as previously described [17]. Each sample was tested in triplicate and was considered to be positive if at least two of the three tests revealed the presence of both genes. The levels of detection of the test were 16 genome copies. To maximize the sensitivity, no internal amplification control was used in the reaction, but there was an external control. All of the positive cases were serotyped using 33 primer couples and TaqMan probes chosen on the basis of pneumococcal epidemiology in European countries and grouped into seventeen multiplex reactions [17, 35–37]. Real-Time amplification was performed in 25-μL reactions that contained 2x QuantiFast Multiplex PCR kit (Qiagen S.r.l., Mi, Italy); primers and FAM or HEX labelled probes were used at a concentration of 250 and 150 nM, respectively.

Analytical specificity was pre-evaluated by means of computer-aided analyses using Primer-BLAST (www.ncbi.nlm.nih.gov/tools/primer-blast) and BLAST (www.blast.ncbi.nlm.nih.gov/Blast.cgi) software to compare the sequences with all of the sequences listed under bacteria and Homo sapiens.

### Statistical analysis

The categorical variables were compared between groups of pneumococcal carrier status using contingency table analysis, reporting absolute frequencies and percentages. The numerical variables were summarized using mean and standard deviation.

Univariate and multivariate ORs, together with pertinent 95 % CIs and *p*-values, were calculated using logistic regression models to measure the association between the selected demographic and clinical characteristics and the pneumococcal carrier status. Adjustment was made for a priori defined covariates such as age (i.e., <75 or ≥75 years) and co-morbidities. All of the analyses were two tailed, and *p*-values of 0.05 were considered to be statistically significant. All of the statistical analyses were conducted using R software, version 3.1.1.

## Abbreviations

95 % CI: 95 % confidence interval
NA: not available
OR: odds ratio
IPD: invasive pneumococcal disease
PCR: polymerase chain reaction
PCV7: 7-valent pneumococcal conjugate vaccine
PCV13: 13-valent pneumococcal vaccine
PPV23: 23-valent polysaccharide pneumococcal vaccine
SD: standard deviation
